# Retraction

**DOI:** 10.1002/cam4.4265

**Published:** 2021-09-27

‘MicroRNA‐92a promotes tumor growth and suppresses immune function through activation of MAPK/ERK signaling pathway by inhibiting PTEN in mice bearing U14 cervical cancer,’ by Zeng‐Hui Li, Lei Li, Lin‐Ping Kang, and Yan Wang, *Cancer Medicine* 2018; 7(7): 3118– 3131. The above article, published online on May 11, 2018 in Wiley Online Library (https://onlinelibrary.wiley.com/doi/10.1002/cam4.1329) has been retracted by agreement between the the journal’s Editor in Chief and Wiley Periodicals LLC. The retraction has been agreed following an investigation based on allegations raised by a third party. A detailed investigation revealed that several image elements of the experimental data (figures 2B and 4B) were published elsewhere in a different scientific context. Furthermore, several methodical flaws and inconsistencies were found. Thus, the editor considers the conclusions of this article to be invalid. The authors were not available to comment on these inconsistencies.

